# 2011–12 Seasonal Influenza Vaccines Effectiveness against Confirmed A(H3N2) Influenza Hospitalisation: Pooled Analysis from a European Network of Hospitals. A Pilot Study

**DOI:** 10.1371/journal.pone.0059681

**Published:** 2013-04-02

**Authors:** Marc Rondy, Joan Puig-Barbera, Odile Launay, Xavier Duval, Jesús Castilla, Marcela Guevara, Simona Costanzo, Katleen de Gaetano Donati, Alain Moren

**Affiliations:** 1 EpiConcept, Paris, France; 2 Centro Superior de Investigación en Salud Pública (CSISP), Valencia, Spain; 3 Centro de Salud Pública de Castellón, Castellón, Spain; 4 Institut national de la santé et de la recherche médicale (Inserm), Réseau National d’Investigation Clinique en Vaccinologie (REIVAC), Paris, France; 5 Université Paris Descartes, Paris, France; 6 Inserm, Centre d’investigation Clinique (CIC) de Vaccinologie Cochin Pasteur BT505, Paris, France; 7 Assistance Publique-Hôpitaux de Paris (AP-HP), Hôpital Cochin, Paris, France; 8 Inserm, CIC 007; AP-HP, Hôpital Bichat Claude Bernard, Paris, France; 9 Instituto de Salud Pública de Navarra, Pamplona, Spain; 10 Centros de Investigación Biomédica en Red (CIBER), Epidemiología y Salud Pública, Barcelona, Spain; 11 Laboratory of Genetic and Environmental Epidemiology, Research Laboratories, Fondazione di Ricerca e Cura “Giovanni Paolo II”, Campobasso, Italy; 12 Department of Infectious Disease, Catholic University, Rome, Italy; University of Hong Kong, Hong Kong

## Abstract

**Background:**

Influenza vaccination strategies aim at protecting high-risk population from severe outcomes. Estimating the effectiveness of seasonal vaccines against influenza related hospitalisation is important to guide these strategies. Large sample size is needed to have precise estimate of influenza vaccine effectiveness (IVE) against severe outcomes. We assessed the feasibility of measuring seasonal IVE against hospitalisation with laboratory confirmed influenza through a network of 21 hospitals in the European Union.

**Methods:**

We conducted a multicentre study in France (seven hospitals), Italy (one hospital), and Navarra (four hospitals) and Valencia (nine hospitals) regions in Spain. All ≥18 years hospitalised patients presenting an influenza-like illness within seven days were swabbed. Cases were patients RT-PCR positive for influenza A (H3N2); controls were patients negative for any influenza virus. Using logistic regression with study site as a fixed effect we calculated IVE adjusted for potential confounders. We restricted the analyses to those swabbed within four days.

**Results:**

We included, 375 A(H3N2) cases and 770 controls. The overall adjusted IVE was 24.9% (95%CI–1.8;44.6). Among the target group for vaccination (N = 1058) the adjusted IVE was 28.8% (95%CI:2.8;47.9); it was respectively 36.8% (95%CI:−48.8; 73.1), 42.6% (95%CI:−16.5;71.7), 17.8%(95%CI:−40.8; 52.1) and 37.5% (95%CI:−22.8;68.2) in the age groups 18–64, 65–74, 75–84 and more than 84 years.

**Discussion:**

Estimation of IVE based on the pooling of data obtained through a European network of hospitals was feasible. Our results suggest a low IVE against hospitalised confirmed influenza in 2011–12. The low IVE may be explained by a poor immune response in the high-risk population, imperfect match between vaccine and circulating strain or waning immunity due to a late season. Increased sample size within this network would allow more precise estimates and stratification of the IVE by time since vaccination and vaccine types or brands.

## Introduction

Worldwide, influenza annual epidemics result in three to five million cases of severe illness and an estimated 250,000 to 500,000 deaths [1]. The average annual rate of influenza-associated hospitalisations was estimated to be between 136 and 309 per 100,000 persons in those aged 65 years and older in the US and England [2]–[4]. As a consequence of the ageing of the population, the overall influenza-related hospitalisation rate tends to increase with time [5]. In Europe, influenza ranks third in terms of number of years of life lost due to mortality from infectious diseases [6].

Measuring influenza vaccine effectiveness (IVE) against severe outcome among at-risk individuals is necessary to guide vaccination strategies. Yet, weak evidence supports their effectiveness in preventing influenza-related morbidity in elderly [7], [8]. Yearly measures of IVE among the most susceptible population may help evaluating the benefit of vaccination programs. Results can also catalyse the research on the development of more immunogenic vaccines for elderly people, the use of larger doses of antigens or the use of antiviral in a more aggressive manner for treatment and prophylaxis. These IVE measures could also lead to recommendations aiming at indirectly protecting elderly people through increased vaccination of transmitter populations or changing the recommendations for the use of the vaccines in terms of timing and targeted population.

With the project “Monitoring vaccine effectiveness during seasonal and pandemic influenza in Europe” (named I-MOVE), the European Centre for Disease Prevention and Control (ECDC) developed a network of study centres in European Union member states measuring seasonal and pandemic influenza vaccine effectiveness against laboratory confirmed medically attended influenza like illness (ILI) during the seasons 2008–2009 through 2012–13[9]–[14]. Beside the Navarra electronic cohort study [15], the I-MOVE network does not allow measuring IVE against severe outcomes.

To measure IVE against severe outcome and to broadly capture a population belonging to the target group for vaccination, laboratory confirmed influenza hospitalisation appeared as an appropriate outcome [7].

In January 2010 the ECDC organised a meeting with potential partners to set up a multicentre hospital based study in EU. This resulted in developing a generic study protocol [16]. In 2011, we launched a pilot study in Spain, France and Italy to estimate the IVE against laboratory confirmed influenza hospitalisation. Sources of funding of study sites and coordination included public and private sectors.

The objective of this project was to assess the feasibility of measuring seasonal IVE against hospitalisation with laboratory-confirmed influenza through a network of hospitals in Europe.

## Materials and Methods

We conducted a multicentre case control study using the test-negative design [17] in 21 hospitals located in France (seven hospitals), Italy (one hospital), and Spain, in Valencia (nine hospitals) and Navarra (four hospitals). Study sites adapted the generic study protocol to the local settings. In each study site, the study period lasted from the week of the first laboratory confirmed case of A(H3N2) influenza until the week of the last laboratory confirmed case of A(H3N2) influenza.

The protocol was approved by the competent Authorities of each country/provinces. The Ethical Principles for Medical Research Involving Human Participants of the World Medical Association and the Declaration of Helsinki (World Medical Association, Inc. Available at: http://www.wma.net/en/30publications/10policies/b3/index.html) were adhered to. According to country specific requirements for ethical approval, all participants (or their legal tutor) provided written consent for recruitment to the study.

The following ethics committees/institutional review boards gave their approval:

- the “Ile de France IV” Ethics Committee (“Comité de Protection des Personnes Ile-de-France IV”), Paris, France
- the Ethical Committee of the Catholic University of Rome, Italy
- the Navarre Ethical Committee for Medical Research, Spain
- the Public Health CSISP Research Ethics Committee of Valencia, Spain.

### Study Population

The study population corresponded to all non institutionalised adults (18 years or older) hospitalised for at least 24 hours in one of the participating hospitals, with no contra-indication for influenza vaccination and onset of influenza-like-illness (ILI) within seven days prior to naso-pharyngeal swabbing. We defined ILI as the presence of at least one systemic symptom (fever, malaise, headache or myalgia) and at least one respiratory symptom (cough, sore throat or shortness of breath). Patients were screened for presence of ILI within the past seven days. In 16 hospitals, this screening applied to patients admitted at the emergency department for a range of pre-defined chief complaints (Table 1). In the other five hospitals, all patients admitted in the participating services were screened.

**Table 1:** Participating services, screening procedure and number of patients screened and included per study site, hospital based Influenza VE study, EU, 2011–12.

Participating hospitals	Participating service(s)	Screening filter	Weekly averagenumber ofadmissions	Reported numberof patientsscreened	Number ofpatients includedin the analysis	Proportion ofpatients includedamong those screened	Patients swabbedwithin 4 days (% ofpatients included)
**France**							
Cochin hospital, Paris	Pneumology, internal medecine	None	33	74	35	47.3%	25 (71,4%)
Bichat hospital, Paris	Pneumology, internal medecine, infectious diseases, gerontology	None	105	76	40	52.6%	28 (70,0%)
Clermont-Ferrand hospital	Intensive care, infectious diseases	None	23	83	14	16.9%	5 (35,7%)
St Eloi hospital, Montpellier	Infectious diseases department internal medicine	Respiratory syndroms	35	196	16	8.2%	8 (50,0%)
St Etienne hospital	Emercency Ward, Pneumoology, Infectious diseases	None	NA	38	9	23.7%	7 (77,8%)
Limoges hospital	Emercency Ward	Respiratory syndroms	64	118	26	22.0%	18 (69,2%)
Rennes hospital	Infectious diseases, pneumology	None	48	62	18	29.0%	9 (50,0%)
**Total - France**			**308**	**647**	**158**	**24.4%**	**100 (63,3%)**
**Navarre**							
Hospital de Navarra	All	Respiratory syndroms	344	64	15	23.4%	11 (73,3%)
Hospital Virgen del Camino			439	180	24	13.3%	17 (70,8%)
Hospital García Orcoyen de Estella			94	5	4	80.0%	4 (100,0%)
Hospital Reina Sofía de Tudela			158	9	1	11.1%	0 (0,0%)
**Total - Navarre**			**1035**	**258**	**44**	**17.1%**	**32 (72,7%)**
**Valencia**							
Hospital de la Plana	Emercency Ward	Respiratory and cardio vascular syndroms	204	667	157	23.5%	100 (63,7%)
Hospital Arnau de Vilanova			211	1 009	179	17.7%	100 (55,9%)
Hospital Pesset			397	1 482	256	17.3%	159 (62,1%)
Hospital San Juan de Alicante			261	733	133	18.1%	88 (66,2%)
Hospital general de Elda			273	981	244	24.9%	181 (74,2%)
Hospital general de Castellon			324	1 222	226	18.5%	130 (57,5%)
Hospital de La Fe			520	787	105	13.3%	62 (59,0%)
Hospital de Xativa			165	423	161	38.1%	65 (40,4%)
Hospital General de Alicante			415	828	207	25.0%	112 (54,1%)
**Total - Valencia**			**2 770**	**8 132**	**1 668**	**20.5%**	**997 (59,8%)**
**Italy (one hospital)**							
**Catholic University hospital**	Emercency Ward	Respiratory syndroms	**630**	**360** *	**25**	**6.9%**	**16 (64,0%)**

* Based on weekly average number of patients admitted with respiratory syndromes.

Patients who had previously tested positive for influenza virus in the 2011/12 season or had received antiviral treatment between the symptom onset and the swabbing were excluded from the study.

All eligible patients who agreed to participated were swabbed and interviewed.

### Data Collection

The swabbing was performed by the hospital physicians in all study sites but Valencia where it was under the responsibility of dedicated study nurses. Data collected included demographics, information on the ILI episode (dates of symptom onset, hospitalisation, laboratory testing and swabbing and treatment), presence of chronic diseases, number of hospital admissions in the past 12 months, number of GP consultations in the previous three months, smoking status, vaccination against influenza in 2011–12 and in the last two seasons and for those aged 65 years and older functional status before onset using the Barthel score [18]. Individuals belonged to the target group for vaccination if they corresponded to the country specific recommendations for vaccination [19]–[21]. Patients were considered vaccinated if they had received a dose of the 2011–12 seasonal vaccine more than 14 days before the date of onset of ILI symptoms. They were considered as unvaccinated if they had received no vaccine or if the vaccine was given less than 15 days before the onset of ILI symptoms.

Data sources included hospital medical records, interview with patient, patient’s family and patient’s physician, vaccination registries and laboratory databases. Vaccination status was ascertained using registries in Valencia and Navarra, interview with patients in France and interview with patients and with their physician in Italy.

### Laboratory Confirmation

Influenza laboratory confirmation was done through reverse transcription polymerase chain reaction (RT-PCR) on nasopharyngeal swabs. Isolates underwent a molecular analysis for currently circulating influenza A viruses (subtypes H3 and H1), A(H1N1)pdm09 and influenza B. In view of the dominance of Influenza H3N2 during the 2011–12 season [22], we restricted the case definition to those patients with a nasopharyngeal sample positive for influenza A(H3N2). Controls were patients with negative samples for any influenza virus.

### Data Management and Analysis

Study sites transmitted anonymised datasets to EpiConcept, the pooled analysis coordinator, through a secured web based system. We ran a complete case analysis, excluding records for which outcome, exposure or confounding variables were missing.

To minimise potential misclassification, we restricted our analysis to those patients swabbed within four days after onset of ILI symptoms. We then ran a sensitivity analysis on all patients swabbed within seven days.

Baseline characteristics of cases and controls were compared using the chi-square test, Fisher’s exact test, t-test or the Mann-Whitney test (depending on the nature of the variable and the sample size).

We assessed qualitative heterogeneity of the studies through site visits to document the recruitment approaches and the strategies set up to ensure the systematic screening and inclusion of ILI patients. We collected information on the vaccines used in the areas covered by the study sites.

We aimed at testing statistical heterogeneity between studies using Cochran’s Q-test and the I^2^ index [23]. We estimated the pooled IVE as 1- the odds ratio (OR)x100, using a one-stage method with study site as a fixed effect in the model. To estimate adjusted IVE, we used a logistic regression model including potential confounding factors: time of symptom onset (by pair of onset weeks), age group (four categories), gender, number of GP visits in the previous three months (more than one vs. one or less), hospitalisation in the previous 12 months, presence of chronic conditions, presence of lung disease and presence of cardiovascular disease.

We stratified IVE in four age groups (18–64, 65–74, 75–84 and 85 years and above) and we confined the analysis to the patients belonging to the target group for vaccination.

We conducted all statistical analysis using Stata version 11 *(StataCorp. 2009. Stata Statistical Software: Release 11. College Station, TX: StataCorp LP).*

## Results

Overall, 9,397 patients were screened in the various hospitals (Table 1). Valencia screened (N = 8,132) and recruited (N = 1,668) the largest number of patients included in this analysis Overall, 8,497 records were received in the pooled database. Of these records, 1,264 were outside the study period, 2,131 were younger than 18 years old or had no age information, 2,171 did not meet the ILI case definition and 986 had been swabbed more than seven days after their symptoms onset. We excluded 10 records because of missing information (vaccination status (6), hospitalisation in the previous year (3) and cardiovascular disease (1)). Eleven patients tested positive for Influenza B, eight for Influenza A(H1N1) and the subtyping was inconclusive for six specimens of Influenza A. These 25 patients were excluded from the analysis. The proportion of patients included among those screened ranged from 6.9% in Italy to 24.4% in France (Table 1). Overall, 1,895 patients swabbed within seven days after illness onset were eligible, including 593 A(H3N2) cases and 1302 negative controls. We restricted our analysis to the 375 cases (63.2%) and 770 controls (59.1%) swabbed within 4 days.

**Figure 1:**
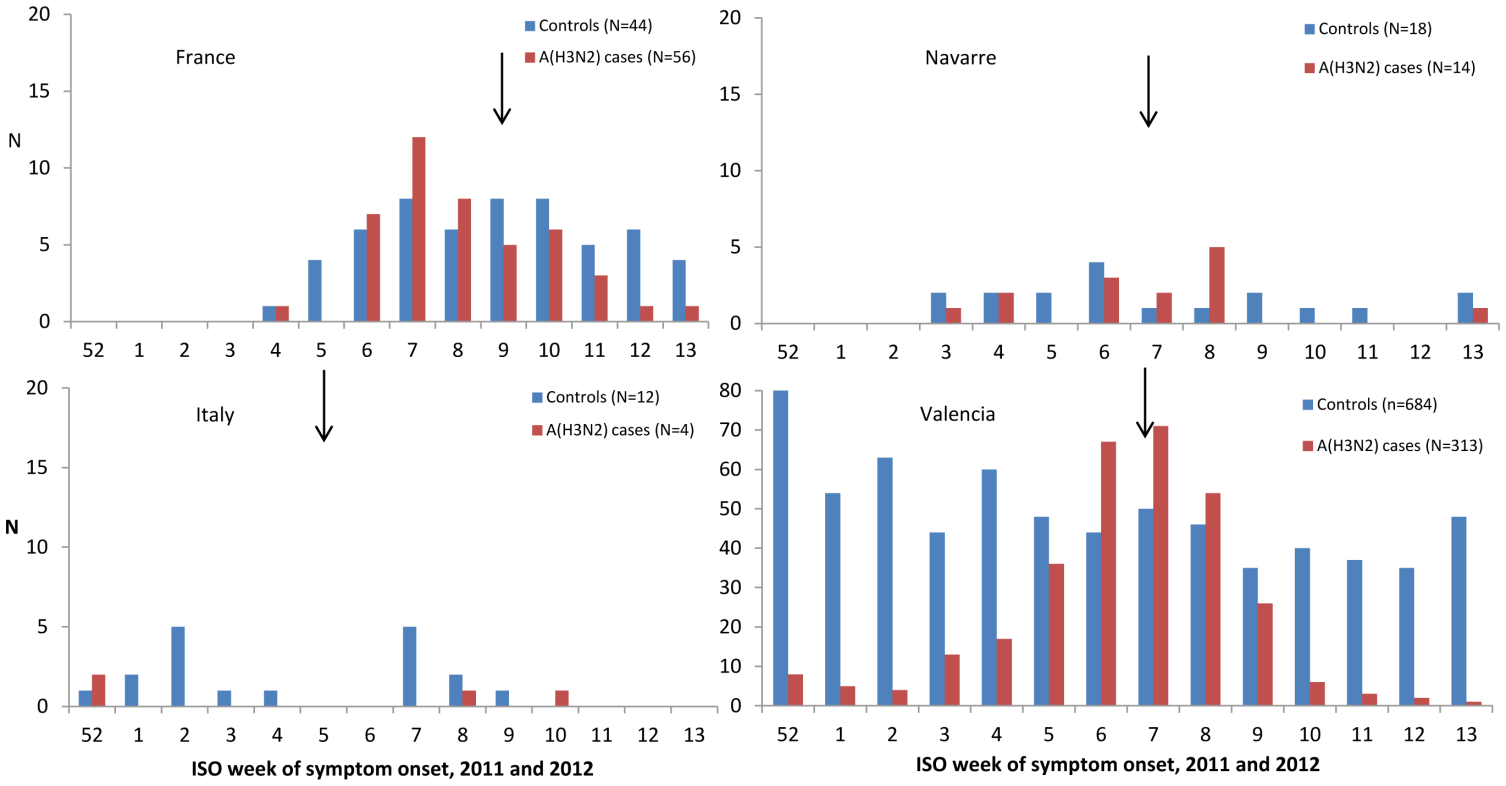
Number of ILI patients positive for influenza A(H3N2) and negative for any influenza by week of symptom onset, hospital based study, and week of peak of influenza activities (pointed by the arrow) in the region. By study site, 2011–12.

**Figure 2:**
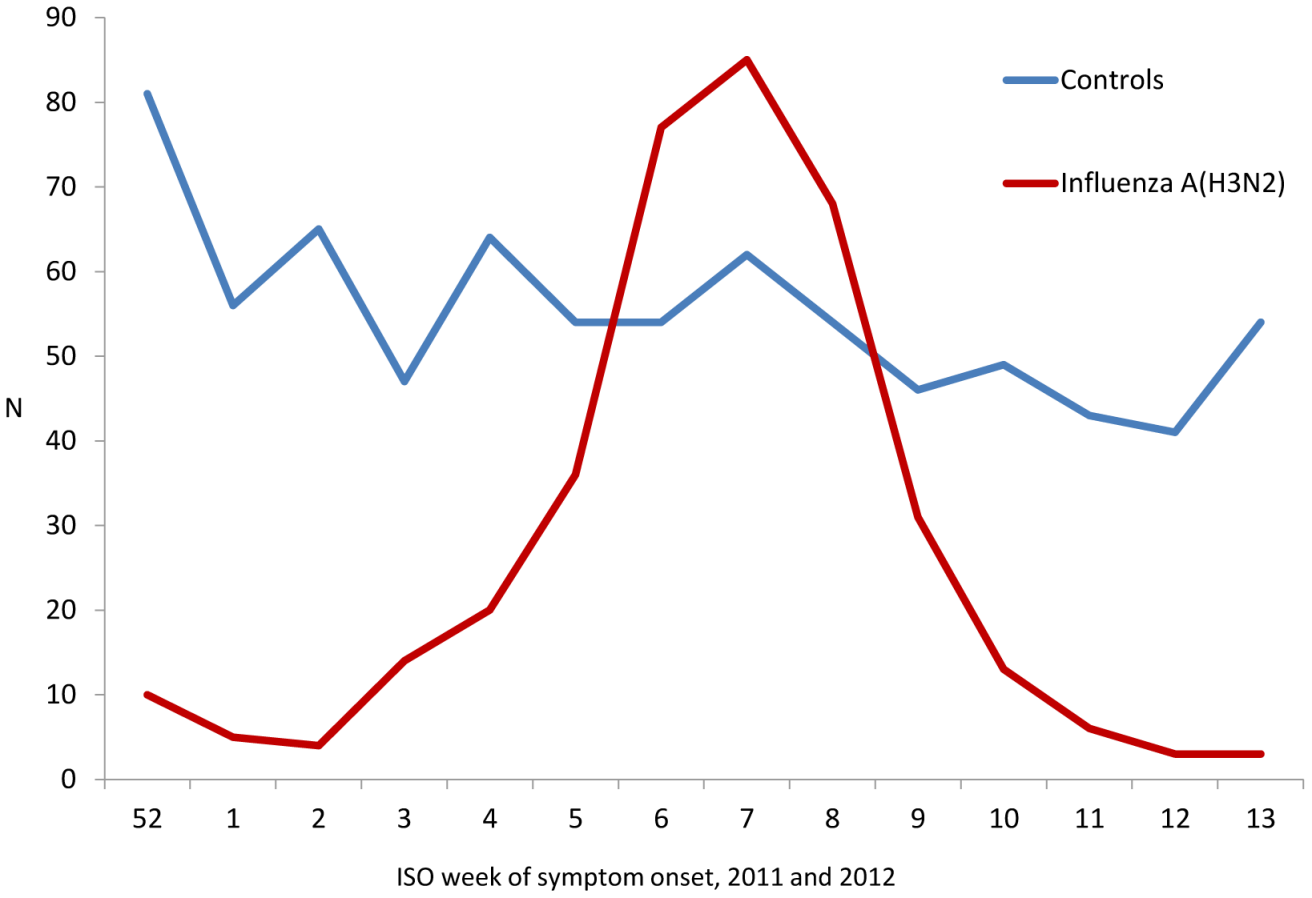
Number of ILI patients positive for influenza A(H3N2) (N = 375) and negative for any influenza (N = 770) by week of symptom onset, hospital based IVE studies, EU – 2011–12.

Based on influenza activities reported through GP sentinel network, the influenza season was earlier in Spain and Italy compared to France [24]–[27]. The inclusions per study site followed the same pattern (Figure 1). In the pooled data, inclusion of cases was highest between the weeks 6 and 8 (Figure 2).

The 2011–12 seasonal influenza vaccination coverage was 54.9% among cases and 59.7% among controls (p = 0.126). The age distribution was not statistically different between cases and controls (p = 0.148) (Table 2). Respectively 93.1% of proportion of cases and 92.1% of controls belonged to the target group for vaccination (p = 0.635). Compared to controls, a larger proportion of cases had cough and fever (p<0.001). The proportion of patients hospitalised in the previous year was 33.9% among cases and 37.4% among controls (p = 0.266). Among the 814 patients aged over 65 years for whom the Barthel score was assessed, a higher proportion of controls than cases had a low functional status (p = 0.023) (Table 2).

**Table 2:** Characteristics of A(H3N2) influenza cases (N = 375) and test-negative controls (N = 770) swabbed less than five days after ILI symptoms onset included in the study, hospital based Influenza VE study, EU, 2011–12.

	Cases	Controls	
	N *(%)*	N *(%)*	p-value‡
**Age group**			
18–64 years	80 *(21.3)*	191 *(24.8)*	0.148
65–74 years	69 *(18.4)*	153 *(19.9)*	
75–84 years	145 *(38.7)*	245 *(31.8)*	
85 years+	81 *(21.6)*	181 *(23.5)*	
**Sex = Male**	213 *(56.8)*	432 (56.1)	0.849
**Belongs to target group for vaccination**	349 *(93.1)*	709 *(92.1)*	0.635
**Symptoms**			
Fever	333 *(88.8)*	616 (80.0)	<0.001
Malaise or headache	278 *(74.1)*	570 *(74.0)*	1.000
Myalgia	74 *(19.8)*	124 *(16.1)*	0.134
Cough	342 *(91.2)*	643 *(83.5)*	<0.001
Sore throat	113 *(30.1)*	223 *(29.0)*	0.588
Shortness of breath	319 *(85.1)*	680 *(88.3)*	0.274
Sudden onset	238 *(64.3)*	495 *(64.5)*	0.947
**At least one chronic condition**	315 *(84.0)*	657 *(85.3)*	0.598
**More than one chronic condition**	187 *(49.9)*	427 *(55.5)*	0.077
**Chronic conditions**			
Diabetes	108 *(28.8)*	216 *(28.1)*	0.834
Heart disease	167 *(44.5)*	362 *(47.0)*	0.449
Lung disease	172 *(45.9)*	384 *(49.9)*	0.620
Immunocompromised	18 *(4.8)*	38 *(4.9)*	1.000
Obese	86 *(22.9)*	228 *(29.6)*	0.020
**More than one GP visit in previous 3 months**	191 *(50.9)*	426 *(55.3)*	0.165
**At least one hospitalisation in previous 12 months**	127 *(33.9)*	288 *(37.4)*	0.266
**Low functional status (among >65 years)** *	50 *(16.9)*	137 *(23.7)*	0.023
**Number of days between onset of symptoms and swabbing**			
0–2 days	105 *(28.0)*	295 *(38.3)*	<0.001
3–4 days	270 *(72.0)*	475 *(61.7)*	
**2011–12 seasonal flu vaccination**	206 *(54.9)*	460 *(59.7)*	0.126
**2010–11 seasonal flu vaccination**	240 *(64.0)*	509 *(66.1)*	0.508

* N = 814 (one record with missing information).

‡ Two-sided Fisher’s exact test.

Site visits and protocol review ensured a homogeneous implementation of the protocol within the participating hospitals. All hospitals systematically included all patients with ILI. In Valencia, dedicated study nurses screened patients for ILI and swabbed them. Elsewhere, clinicians swabbed the patients. Due to low number of sites and small sample size, the statistical heterogeneity could not be assessed.

The overall crude IVE (N = 1145) was 23.1% (95% CI: −1.5; 41.8). The adjusted IVE was 24.9% (95% CI: −1.8; 44.6). Among those aged less than 65 years (N = 271), the adjusted IVE was 16.0% (95% CI: −73.0; 59.2).

Among the target group for vaccination (N = 1058) the adjusted IVE was 28.8% (95% CI: 2.8; 47.9). The adjusted IVE were respectively 36.8% (95% CI: −48.3; 73.1), 42.6%(95% CI: −16.5; 71.7), 17.8% (95% CI: −40.8; 52.1) and 37.5% (95% CI: −22.8; 68.2) in the age groups 18–64, 65–74, 75–84 and more than 84 years (Table 3).

**Table 3:** Pooled crude and adjusted Influenza vaccine effectiveness against influenza A(H3N2) in target group for vaccination swabbed less than five days after ILI symptoms onset (N = 1058), by age group, EU, 2011–12.

				Percent vaccinated (%)			
Population	Model used	N	Number of cases	cases	controls	IVE (%)	95% CI
**All target population** a	Crude*	1058	349	57.6	63.5	30.4	6.6; 48.1
	Adjusted					28.8	2.8; 47.9
**Age group** b							
18–64 years^ 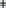 ^	Crude*	160	54	31.5	40.6	44.1	−23.2; 74.6
	Adjusted					36.8	−48.3; 73.1
65–74 years¥	Crude*	205	69	52.2	58.8	37.9	−20.3; 67.9
	Adjusted					42.6	−16.5; 71.7
75–84 years±	Crude*	389	145	66.9	72.1	23.4	−28.1; 54.3
	Adjusted					17.8	−40.8; 52.1
85 years and olderΩ	Crude*	244	80	63.8	73.2	39.6	−15.9; 68.5
	Adjusted					37.5	−22.8; 68.2

* Adjusted for study site and week of onset.

a Adjusted for study site, week of symptoms onset, age group (four categories), gender, GP visit in the previous three months, hospitalisation in the previous year, presence of chronic condition, presence of lung diseases and presence of cardiovascular disease.

b Adjusted for study site, week of symptoms onset, gender, GP visit in the previous three months, hospitalisation in the previous year, presence of chronic condition, presence of lung diseases and presence of cardiovascular disease.

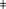
24 controls dropped due to no cases in this age group, targeted by the vaccination, on the 7th pair of weeks.

¥ 16 controls dropped due to no cases in this age group on the 7th pair of weeks. 1 control dropped due to no cases in Italy.

± 1 control dropped due to no cases in Italy.

Ω 17 controls dropped due to no cases in this age group on the 7th pair of weeks. 1 case dropped due to no controls in Italy.

When we included patients swabbed 5 to 7 days after ILI onset, the overall adjusted IVE (N = 1895) was 17.5% (95% CI: −4.4; 34.7) and 20.7% (95% CI: −0.6; 37.6) among the target group for vaccination (N = 1754).

## Discussion

The results of our 2011–12 pilot multicentre hospital based influenza study suggest a low IVE against laboratory confirmed A(H3N2) Influenza. The IVE point estimate was 24.9% overall and ranged between 17.8% in the 75–84 years and 42.6% in the 65–74 years.

The 21 hospitals followed a common core protocol, allowing for pooling data sets. The systematic inclusion of ILI patients and the access to medical records ensured the collection of a good quality data. We had very few missing values (0.5%) and were able to perform a complete case analysis. We used RT-PCR confirmation allowing measuring IVE against a very specific outcome [28]. The absolute difference between crude and adjusted IVE varied from 1.6% (among all targeted population) to 7.3% (among the less than 65 years targeted by the vaccination) suggesting little confounding from the variables included in our study.

Ninety two percent of the ILI patients belonged to the target groups for vaccination. Estimating effectiveness against laboratory confirmed influenza in this population is particularly relevant since no efficacy measures are available [7].

Some biases may limit the interpretation of our study. High risk groups are more likely to be vaccinated and to develop a severe form of influenza. This may overestimate the number of vaccinated cases seen at the hospital and underestimate IVE. People with a healthy lifestyle are more likely to accept/request vaccination and less likely to be severely sick. This would overestimate IVE. However, while this bias is likely to happen for mild outcomes, it is unlikely to affect the IVE estimate in a hospital setting. Extremely frail people are less likely to be offered vaccination but more likely to develop a severe form of the disease. This would overestimate IVE [29]. We collected detailed information on severity of chronic conditions and functional status. This allowed us to correct for this potential confounding. However, we cannot exclude that residual confounding still biases our results.

Over a third of patients included in this study were swabbed between five and seven days after onset of symptoms. Although hospitalised patients were described as shedding influenza virus for longer periods [30], the likelihood of misclassifying patients’ outcome increases with time. To reduce the chance for misclassification bias, we restricted our analysis to those swabbed within four days. We are confident that, by adopting this approach, we limited the presence of false negatives in our study population. In the sensitivity analysis including all patients swabbed within seven days, the IVE was lower suggesting the presence of misclassification biases. In the future, further studies investigating the duration of shedding of seasonal influenza viruses among high risk population would help setting up cut-offs to reduce misclassification biases.

The sources of information included medical records consultation and results of very specific laboratory tests, minimising information biases. Vaccination status ascertainment relied on registries in the Spanish studies, patients’ interview in France and Italy, with confirmation of information by the practitioner in Italy. At the time of interview, patients did not know if they had confirmed influenza. This limited differential recall of vaccination status between cases and controls.

The source population giving rise to the cases can be defined as individuals likely to be hospitalised in case of severe ILI. Considering the good access to hospital care in France, Spain and Italy, the source population can be defined as the general population. Recruiting controls in the community would be logistically challenging as a very large sample size would be needed to adjust for the numerous potential confounders. Recruiting controls among those hospitalised for ILI testing negative for influenza has the advantage of being resource saving as it does not require extra sampling. However, test negative controls may not be representing the vaccination coverage in the general population. In our analysis, vaccination coverage among controls was 59.7% and 63.5% among those belonging to the target group for vaccination. A population based study estimated the vaccination coverage in France to be 23% in the general population aged 15 years and older in 2011–12 [31]. During this season, the vaccine coverage was 59% among non institutionalised targeted population in Navarre [26] and 49% among targeted population in France [31]. The observed vaccine coverage in our control groups is close to the coverage reported among the target group for influenza vaccination. Furthermore, 92.1% of the controls belonged to the target group for vaccination. Selection biases are certainly minimised in our analysis confined to the target group for vaccination.

Sample size varied across study sites, ranging from 25 patients in Italy to 1668 in Valencia region. The performance of a one-stage pooled analysis also assumes that the IVE and confounding are similar in all studies. Considering the broad range of vaccines used across sites (18 vaccine brand names) and potential differences in health care use, we can expect IVE and confounding effects to vary across study sites. If so, a two-stage model and larger sample sizes in each study site are needed.

Considering the lack of power to assess statistical heterogeneity across study sites [23], qualitative heterogeneity assessment is of great relevance in this analysis. We conducted site visits and documented the protocol implementation within each hospital to assure systematic recruitment processes and provide recommendations if needed. The four sites had a different research status impacting on patients’ recruitment. In Navarra region, health data are computerised and a systematic swabbing of ILI patients is implemented in the hospitals. France and Italy implemented this pilot protocol as non-interventional studies. Swabbing had to be part of the usual patient management and was the responsibility of clinicians. As a consequence most Italian and French hospitals had difficulties to comply with an exhaustive swabbing of ILI patients. In Valencia, one study nurse was hired in each hospital and was in charge of the recruitment and the swabbing of all patients with ILI in the past seven days. This active surveillance and swabbing of eligible patients, conducted independently from the routine case management, seems crucial to ensure a systematic inclusion of all hospitalised ILI patients from the source population and reach large sample size.

Our study suggests a low VE against laboratory confirmed influenza A(H3N2) hospitalisation in the 2011–12 season. Our estimates are lower than the previously published results from IVE against GP attended influenza [10], [26], [32] among vaccination target groups this season. Our study population was older and more likely to have at least one chronic condition compared to GP based populations. Lower effectiveness and efficacy of influenza vaccines among the elderly can be explained by a lower immune response [26], [27]. These observations, underline the need of developing more immunogenic vaccine formulations for the elderly.

In 2011–12, the influenza A(H3) virus circulating has moved genetically and antigenically away from seasonal vaccine viruses [33], [34]. In addition, the 2011–12 season occurred very late compared to previous seasons. The time lag between the vaccination campaigns and the beginning of the epidemics was longer than usual. Protection against vaccine strains begins within two weeks of immunisation, peaks at 4–6 weeks and then wanes [35]. A waned protection could partially explain this low VE, as discussed in recently published papers [36]–[38]. Bigger sample sizes are needed to measure IVE against hospitalised Influenza according to time since vaccination.

This study allowed the collection of good quality data. Patients belonging to the target group for vaccination are an appropriate study population to guide influenza vaccination strategies in Europe. To compute more precise IVE and be able to estimate specific IVE by vaccine type and mode of administration, increasing the samples within hospitals are needed to better assess the quantitative validity of the pooling of data. Maintaining harmonised practices across study sites through a continuous and strong coordination will ensure the qualitative validity of the pooling. Our pilot study suggests that a multicentre hospital based study is feasible and needed in EU to measure IVE against hospitalised influenza. A large hospital study network in EU could allow for studying VE against various vaccine preventable diseases while optimising the high cost of such a network.
